# *CmYAB2* transcriptional module regulates fruit morphogenesis in *Cucumis Melo*

**DOI:** 10.1186/s12870-026-09107-3

**Published:** 2026-06-08

**Authors:** Yanfang Bao, Wenjing Zhang, Chenchen Jia, Xiao Zhang, Agula Hasi, Gen Che

**Affiliations:** Key Laboratory of Herbage & Endemic Crop Biology, Ministry of Education, School of Life Sciences, Inner Mongolia University, Hohhot, 010070 China

**Keywords:** Melon, YABBY family, *CmYAB2*, Expression profiling, Fruit size, Molecular mechanism

## Abstract

Melon (*Cucumis melo* L.) is a globally important horticultural crop valued for its favorable qualities and economic value. The YABBY transcription factor family, known for dual activator/repressor functions in regulating secondary metabolites and developmental processes, remains functionally uncharacterized in melon. Here, we identified seven *CmYAB* proteins containing conserved C2C2 zinc-finger and YABBY domains. Transcriptome profiling and RT-qPCR results revealed specific expression of *CmYAB2* in the melon bisexual flowers and developing fruits. We performed melon genetic transformation and found that *CmYAB2* overexpression significantly increased fruit vertical and horizonal diameters. Transcriptome sequencing of transgenic melons suggested that *CmYAB2* affects melon fruit size by mediating cell division/expansion related genes and hormone pathway regulators. Performing yeast two-hybrid (Y2H) and bimolecular fluorescence complementation (BiFC) assays, we revealed the direct protein interaction between CmYAB2 and CmYAB5b transcription factors in plant nucleus. Then, we also found that CmYAB2 protein can directly bind to the promoter of a melon fruit size regulator gene *CmSUN25-26-27c* and inhibits its expression. This work elucidates the role of *CmYAB2* transcriptional module in regulating melon fruit development and provides critical genetic resources for further molecular breeding.

**Supplementary Information:**

The online version contains supplementary material available at 10.1186/s12870-026-09107-3.

## Introduction

Melon (*Cucumis melo* L.), an annual climbing herb of the Cucurbitaceae family, is a globally important horticultural crop [1]. Melon fruits have high vitamin content and sweet taste [2, 3]. Melon has undergone complex domestication, which suggests three independent events yielding in two cultivars of *C.melo* ssp. *melo* and *C.melo* ssp. *agrestis* [4]. Fruit size (FS) critically determines melon morphology, quality, and yield [5, 6]. Domestication has driven a pronounced shift from small, spherical wild fruits to larger, morphologically diverse cultivated varieties [7]. Although quantitative trait locus (QTL) mapping has identified multiple genomic regions governing melon FS and fruit shape index (FSI) [8], the underlying candidate genes and their molecular mechanisms remain largely unknown, awaiting further exploration.

Plants development undergone molecular mechanisms and signaling networks to regulate gene expression and adjust environmental conditions [9]. Fruit development involves coordinated multiple gene families, including the plant-specific YABBY transcription factor family [10, 11]. YABBY family proteins have a conserved YABBY domain at the C-terminal and a zinc finger (C2C2) domain at the N-terminal domain [12]. In the model plant *Arabidopsis*, *YABBY* gene family includes *CRABS CLAW* (*CRC*), *INNER NO OUTER* (*INO*), *YAB2*, *YAB3*, *FILAMENTOUS FLOWER* (*FIL*), and *YAB5*, regulating organ polarity and floral development [13]. The YABBY family transcription factors participate in regulating plant leaf morphogenesis, reproductive development, and abiotic stress responses, exhibits both conserved and divergent functions across species [14–16]. In the monocot plant maize, ZmYABBYs are highly expressed in leaves and shoot apical meristems (SAM) [17]. 8 YABBY family proteins were identified in *Oryza sativa*, with the *CRC* homolog gene *drooping leaf* modulates rice leaf architecture [18]. *OsYABBY1* modulates gibberellin synthesis and is expressed in floral primordia [19, 20]; *OsYABBY2* regulates seed development [21]. In soybean, *GmYABBY10* acts as a negative regulator of drought and salt stress tolerance [16]. In grape, *VvYABBY4* (*YAB2*) transgenic tomatoes exhibit dwarfing and delayed pistil/pollen development [22]. *CaYABBY5* gene is predominantly expressed in the petals and carpels of pepper plants, and its overexpression in tomatoes influences fruit morphogenesis [23]. In dicot plants, CRC homologs are essential for establishing carpel identity, fruit development, and nectary formation. Several studies have underscored their importance across various species [24–28]. Specifically, in the cucumber, a representative of the Cucurbitaceae family, the *CsCRC* gene has been identified as a key regulator that activates the downstream gene *CsARP1*. This activation leads to cell expansion in cucumber fruits, thereby contributing to their elongation [29]. Overall, research highlights the significant influence of genes such as YAB5 and CRC on fruit development, emphasizing their importance in agricultural research and potential applications for improving crop varieties.

Despite extensive profiling in model plants, the function of YABBYs in *Cucumis melo*, particularly their function on fruit development, remains elusive. In this study, we identified seven melon *YABBY* genes (*CmYABs*). Genetic transformation confirmed the role of *CmYAB2* in fruit enlargement. Protein-protein interaction and protein-DNA interaction assays revealed the direct interaction of *CmYAB2* with CmYAB5b protein and *CmSUN25-26-27c* gene. This work elucidates a novel *CmYAB2*-mediated gene regulatory pathway governing melon fruit size.

## Materials and methods

### Identification of YABBY gene family in melon

We searched from *Arabidopsis thaliana* genome database (https://www.arabidopsis.org/) to obtain the AtYABs amino acids. Using Blast in melon genome database (http://www.cucurbitgenomics.org/organism/18) identify melon YABBY family members all candidate. The hidden Markov model file of the YABBY family was constructed by using the Pfam database and the hmm search program in the HMMER3.0 software package. The candidate YABBY proteins were compared and searched to obtain the CmYABs proteins containing the conserved N-terminal C2C2 domain and the C-terminal YABBY domain. We used the ExPASy (http://expasy.org/) online tool to analyze the length of the open reading frame (ORF), the number of amino acids (aa), the molecular weight (MW) and the isoelectric point (pI) of melon YABBYs. WoLF PSORT (https://wolfpsort.hgc.jp/) for subcellular localization prediction tool. When analyzing intraspecific collinearity, we first downloaded the genome sequence file and gene annotation file of the melon database and used TBtools to extract the information from the genome file and GFF file. Using the “One-step MCScanX” module in the TBtools software, we drew the intraspecific collinearity map of the “YABBY”family in melon.

In order to explore the evolutionary relationship of YABBY gene family among *Arabidopsis thaliana*, *Oryza sativa* (https://rice.uga.edu/index.shtml), *Cucumis sativus* (http://www.cucurbitgenomics.org/organism/2), *Solanum lycopersicum* (https://solgenomics.net/) and *Cucumis melo*, we used the Neighbor-Joining method in MEGA software to build the phylogenetic tree, setting the parameters as 1000 repeats. We visualized phylogenetic trees at iTOL online (https://itol.embl.de/). Draw collinearity graphs using TBtools software. Through the MEME online website (https://meme-suite.org/meme/tools/meme) [30], we analyzed the melon YABBY protein conservative motif and obtained the YABBY gene family members base sequence type and the order.

### Chromosome localization and promoter cis-acting element analysis of YABBY gene in melon

According to our identified melon YABBY genome information, melon genome data and MG2C online software (http://mg2c.iask.in/) were used to construct the chromosomal physical map of *YABBY* gene family. We used the melon genome database to obtain the upstream 2000 bp sequence of the melon *YABBY* family genes. PlantCARE website (https://bioinformatics.psb.ugent.be/webtools/plantcare/html/) was used for analyzing promoter regions.

### Plant materials and culture conditions

The Hetao melon inbred line was cultivated in Dengkou County, Bayannur City, Inner Mongolia Autonomous Region. Melon materials were provided by Hasi Laboratory (Inner Mongolia University, Hohhot, China) [31, 32]. We obtained samples of roots, stems, leaves, bisexual flowers, male flowers and ovary from 28-day (4-week-old) seedlings and set up 3 biological replicates for each sample. All tissue samples were rapidly frozen in liquid nitrogen and stored in an ultra-low temperature environment of -80 °C. The seeds of *Nicotiana benthamiana* were stored in our laboratory and cultured in a plant incubator after seeding in a seedling medium.

### Expression profile analysis and identification of transgenic plants

RNA-Seq data was used for displaying the expression heat map through TBtools software [33]. The total RNA of melon tissues (roots, stems, leaves, bisexual flowers, male flowers, ovary and growing fruit) were extracted according to the instructions of TRIzon Reagent. cDNA was obtained using PrimeScript^®^RT reagent Kit and gDNA Eraser reverse transcription Kit and was used as the template for RT-qPCR experiments. Gene expression level was estimated by RT-qPCR using *GAPDH* as reference gene. Three biological replicates and three technical replicates were performed and analyzed by 2^−ΔΔCT^ method. The experimental results were plotted by Graphpad Prism 8 software.

To identify vector-positive T1 plants, seeds were collected from mature melon fruits and sown in seedling trays within an artificial climate chamber. After the plants developed their second to third true leaves, 100 mg of young leaves were harvested for genomic DNA extraction. PCR was then performed to confirm vector-positive plants. These confirmed plants were subsequently transplanted into soil for phenotypic observation. Detection primers were designed based on the resistance gene sequence of the vector using Primer 5.0 software, with the primer sequences provided in Supplemental Table 1. Then, RT-qPCR was conducted to evaluate the gene overexpression levels in these plants.

### Gene cloning and genetic transformation of melon

We used Primer 5.0 software to design *CmYAB2* (*MELO3C014527.2*) genespecific primers, amplified the full-length coding sequence and recombined the target gene into the 35 S promoter-driven overexpression vector pCAMBIA1305. Primers are listed in supplemental Table 1. In order to obtain transgenic plants, the recombinant vector pCAM1305-*CmYAB2* plasmid was diluted to 100 ng/µL with 2 × SSC infusion (sodium citrate buffer, pH = 7.0) and stored at -20℃. Artificial self-pollination was conducted in the melon flowers at anthesis day. Approximately seven hours post-self-pollination, a microsyringe was used to extract 10 µL of the introduction liquid into the style of the bisexual flower. This prepared solution was then meticulously and gradually injected along the pollen tube into two-thirds of the melon ovary [34, 35]. Vector specific primers were designed to conduct PCR positive detection on the T_1_ generation transgenic plants. We obtained 6 lines of *CmYAB2* overexpression melons and selected three stable transgenic lines for phenotype observation.

### Subcellular localization experiment

We used Primer 5.0 software to analyze the cleavage site of *CmYAB2*, removed the stop codon of the *CmYAB2* coding sequence and inserted to the pCAMBIA-1300 vector. In order to analyze the localization of CmYAB2 protein, the recombinant plasmid pCAM-*CmYAB2* and the vector pCAMBIA1300 plasmid were transformed into competent *Agrobacterium* GV3101 cells and were transient expressed in tobacco leaves. The fluorescence signal excited at a wavelength of 488 nm was observed using a laser confocal microscope (NIKON AX confocal laser microscope, Japan). The primer information was listed shown in supplementary Table 1.

### Yeast two-hybrid and BiFC experiments

We constructed the full-length sequences of *CmYAB2* and *CmYAB5b* (*MELO3C004493.2*) into the vector pGADT7 and pGBKT7, respectively. Recombinant vector plasmids pAD-*CmYAB2* and pBK-*CmYAB5b* were transformed into yeast strain AH109 [36] and protein interactions were screened on SD/-Trp/-Leu/-His/-Ade solid medium, and staining was performed using x-α-gal.

Specific primers were designed to amplify the coding sequences of *CmYAB2* and *CmYAB5* genes without stop codon and the target fragments were connected to the pSPYCE-35 S and pSPYNE-35 S vectors, respectively [36]. Recombinant vector plasmids pCE-*CmYAB2*, pNE-*CmYAB5* and empty plasmids pSPYCE-35 S, pSPYNE-35 S were transformed into *Agrobacterium* GV3101 receptive cells. The lower epidermis of tobacco leaves was infected and the interactions in vivo were observed 48 h later using confocal laser microscopy (NIKON AX confocal laser microscope, Japan).

### Yeast one-hybrid and dual-luciferase transcriptional reporter assays

We obtained the cDNA sequence of *CmSUN-25-26-27c* (*MELO3C013004.2*) gene from the melon genome database and extracted the 2000 bp upstream promoter sequence. The cis action elements of the promoter were analyzed using the PlantCARE website, and it was found that the elements GAAAGAA were present. We analyzed the 2000 bp promoter region upstream of the *CmSUN-25-26-27c* gene and subcloned the cis-element (GAAAGAA) into the pAbAi vector. This recombinant plasmid pAbAi-CmSUN25-26-27c was then transformed into Y1H Gold receptor cells. To determine the appropriate inhibitory conditions, we screened for the AbA concentration that completely inhibited the growth of the strain on SD/-Ura solid medium. Subsequently, co-transformed yeast cells were cultured on SD/-Ura/-Leu medium supplemented with 500 mg/L AbA to verify the interaction [37].

We constructed *CmYAB2* sequence and 2000 bp upstream promoter sequence of *CmSUN25-26-27c* gene onto protein expression vector pGreenII-62-SK and bifluorescent vector pGreenII-0800, respectively. The recombinant plasmids 62SK-*CmYAB2*, 0800-*SUN25-26-27c*, empty plasmids pGreenII-62-SK, pGreenII-0800 were transformed into *agrobacterium* GV3101 (pSoup) and injected to tobacco leaves. The leaf samples were treated with a TransDetect kit and the LUC enzyme activity was measured by the LumiPro. The ratio of LUC/REN was calculated after all samples were tested.

### Transcriptome data analysis

Mature fruit pulp samples from wild-type (WT) and *CmYAB2*-overexpression (*CmYAB2*-OE) transgenic plants were harvested for comparative transcriptome analysis. Six biological replicates (3 per genotype) underwent paired-end RNA sequencing (150 bp) on the DNBSEQ-T7 platform (OEBIOTECH Co., Ltd., Shanghai) with a minimum depth of 20 million reads per sample. Raw sequencing data were deposited in the NCBI Sequence Read Archive (PRJNA1282919). Differentially expressed genes (DEGs) were identified using HISAT2 (v2.2.1) alignment to the *Cucumis melo* reference genome (v4.0) and DESeq2 (v1.30.1) analysis (|log₂FC| ≥ 1, FDR < 0.05). Functional annotation of DEGs was performed via Gene Ontology (GO) enrichment and KEGG pathway analysis.

## Results

### *Identification of melon* YABBY *family genes*

**Fig. 1:**
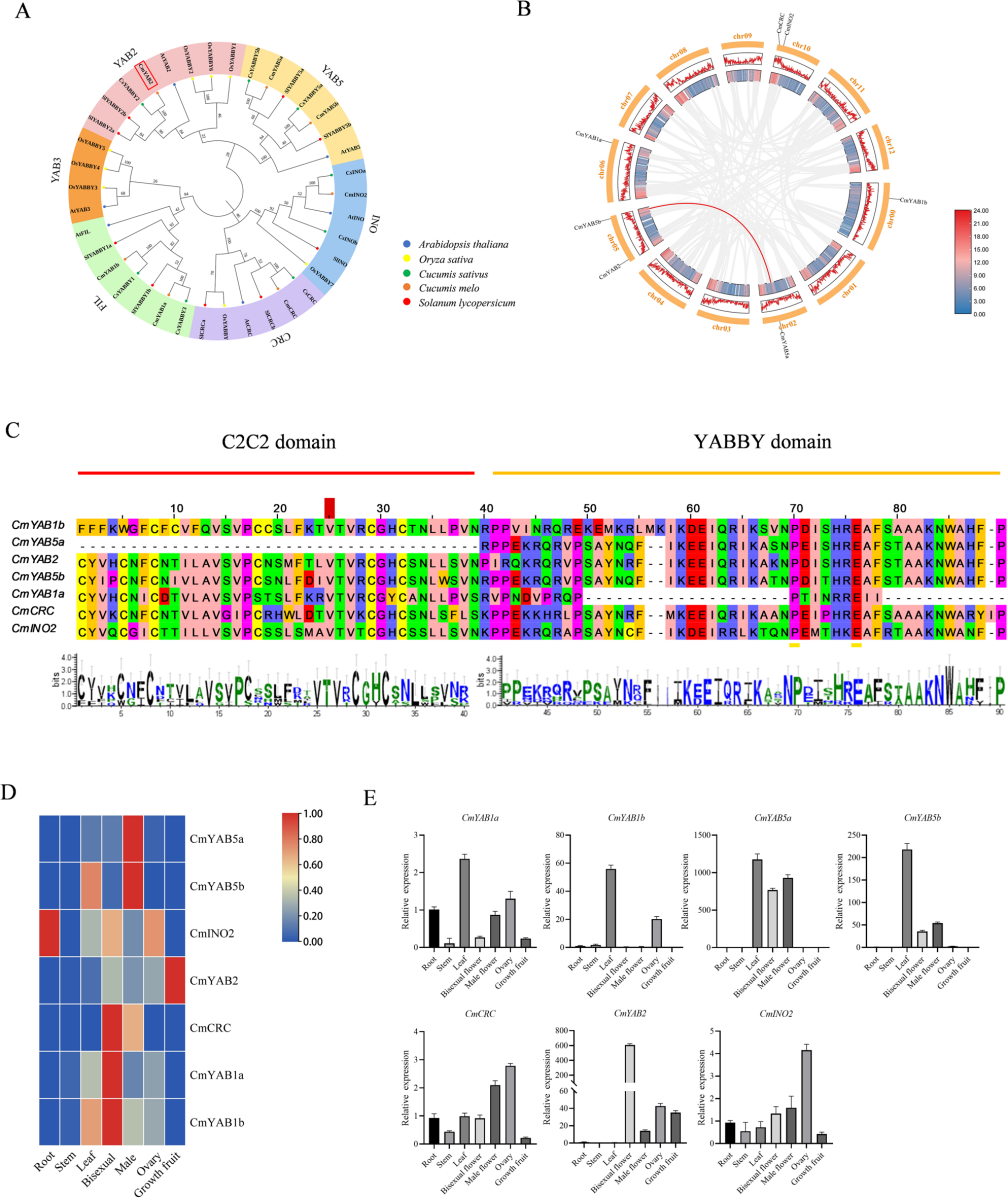
CmYABs sequence analysis and expression profile analysis of the *CmYABs* gene in melon. **A** Phylogenetic trees of YABBY protein in *Arabidopsis thaliana*, *Oryza sativa*, *Cucumis sativus*, *Solanum lycopersicum* and *Cucumis melo*. Different clades represent the six subfamilies. The red box indicates the CmYAB2 gene. **B** Collinearity analysis among *CmYABs* **C** The conserved domains of CmYABs. Zinc finger (C2C2) domain and the YABBY domain were characterized. **D** Heatmap analysis of *CmYABs* expression levels in melon roots, stems, leaves, bisexual flowers, male flowers, ovary and growing fruits. **E** RT-qPCR analysis of *CmYABs* in melon tissues. Three biological replicates and three technical replicates were performed

Using *Arabidopsis thaliana* YABBY sequences as reference, we identified seven YABBY family members in *Cucumis melo*. To elucidate functional relationships, we performed phylogenetic tree analysis of 38 YABBY protein from *Arabidopsis thaliana* [38], *Oryza sativa* [18], *Cucumis sativus* [39], *Solanum lycopersicum* [40] and *Cucumis melo*. Phylogenetic tree revealed six distinct subfamilies (Fig. 1A), with the YAB2 clade containing the highest membership (*n* = 8). CmYAB2 clustered within the YAB2 subfamily alongside AtYAB2. The intraspecific collinearity analysis showed only one collinearity pair of *CmYAB5a* and *CmYAB5b* among melon *CmYABs*, suggesting their relative stable physical positional relationship during evolution (Fig. 1B). We analyzed the conserved domain and confirmed melon YABBY proteins harbor both the N-terminal C2C2 zinc finger and C-terminal YABBY domains (Fig. 1C). Physicochemical characterization revealed melon YABBY proteins range from 133 to 224 amino acids (14.67–25.75 kDa) with isoelectric points (pI) spanning 6.38 (CmYAB1a) to 9.81 (CmYAB1b) (Table S1). Conserved motif analysis using MEME revealed three motifs in CmYABs. CmYAB2, CmYAB5b, CmCRC, and CmINO2 contained all three motifs (Fig. S1A). In CmYAB1a and CmYAB5a, Motif 1 and Motif 2 are absent, respectively. Seven *CmYAB* genes localized to chromosomes 2, 5, 6, and 10 (Fig. S1B). Promoter analysis via PlantCARE identified 10 cis-elements categorized into hormone-responsive (GA, ABA, SA, MeJA), stress-responsive, light-responsive, endosperm development (P-box), and meristem-specific expression (Fig. S1C). All *CmYAB* promoters contained hormone-response elements, indicating potential phytohormone network involvement.

### YABBY gene expression profiling in melon

Transcriptomic profiling across melon tissues (roots, stems, leaves, bisexual/male flowers, ovaries, developing fruits) revealed distinct spatiotemporal expression patterns of *CmYAB* genes (Fig. 1D). *CmYAB1b*, *CmYAB1a*, and *CmCRC* exhibited specific enrichment in bisexual flowers, while *CmYAB2* showed maximal expression in developing fruits. *CmINO2* demonstrated root-specific expression, and the paralogs *CmYAB5a/b* displayed specific expression in male flower with *CmYAB5b* additionally expressed in leaves. Through RT-qPCR, we found that *CmYABs* could be divided into two groups (Fig. 1E): vegetative enriched-genes (*CmYAB1a*, *CmYAB1b*, *CmYAB5a*, and *CmYAB5b*), exhibiting highest expression in leaves. Reproductive-enriched genes (*CmCRC*, *CmYAB2*, and *CmINO2*) showing relative high expression in floral tissues (bisexual/male flowers), ovaries, and developing fruits.

### Overexpression of CmYAB2 gene leads to larger fruit

**Fig. 2:**
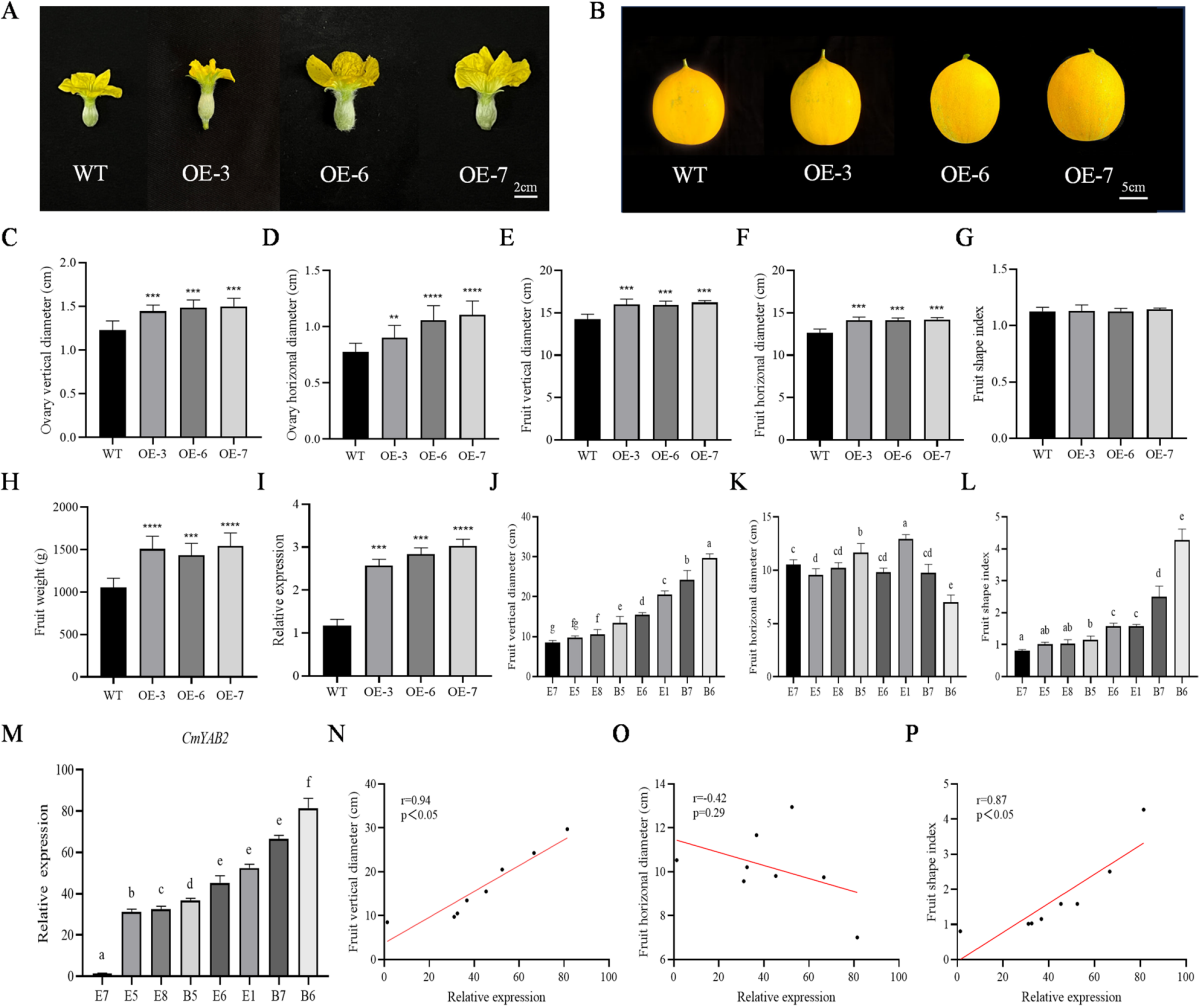
*CmYAB2* positively regulates melon fruit development. **A** Phenotype observation in melon ovary at anthesis day. Scale bar = 2 cm. **B** *CmYAB2*-OE fruits at mature stages enlarged compared to WT. Scale bar = 5 cm. **C** The vertical diameter of the ovary at anthesis day. **D** The horizonal diameter of the ovary at anthesis. **E** The vertical diameter of mature fruits. **F** The horizonal diameter of mature fruits. **G** Fruit type index of mature fruits. **H** The weight of mature fruits. **I** The relative expression level of *CmYAB2* gene in the ovary of WT and transgenic lines. ** *p*-value < 0.01, *** *p*-value < 0.001, **** *p*-value < 0.0001. **J** Vertical diameter, **K** horizonal diameter, and **L** fruit shape index of different melon cultivars. **M** Expression of *CmYAB2* in different melon cultivars. **N** Correlation between *CmYAB2* expression level and vertical diameter, **O** horizonal diameter, and **P** fruit shape index of different melons

To further explore the function of *CmYAB* gene in melon fruit development, we selected *CmYAB2* gene, which has high expression in bisexual flowers and ovaries, and generated transgenic melon lines of its overexpression vector. Three representative transgenic lines of *CmYAB2*-OE (OE-3, OE-6 and OE-7) exhibited significant fruit expansion phenotypes (Fig. 2A-B). At fruit mature stage, *CmYAB2*-OE lines exhibited significantly enlarged fruits compared to WT (Fig. 2B). The statistical analysis showed that vertical and horizonal diameters of the fruits at ovary stage and ripening stage of *CmYAB2-*OE plants are significantly larger than WT (Fig. 2C-F). Horizontal diameters of mature fruits in transgenic melons expanded from 12.64 ± 0.85 cm (WT) to 14.15 ± 0.75 cm (OE-3), 14.14 ± 0.46 cm (OE-6), and 14.20 ± 0.50 cm (OE-7) (average increase: 1.52 ± 0.03 cm; Fig. 2F). The morphological expansion corresponded to substantial weight gains of 379.72–491.97 g per fruit (Fig. 2H) with no significant difference in fruit shape index (Fig. 2G). Consistently, RT-qPCR confirmed more than 2.5-fold higher *CmYAB2* expression in transgenic lines (Fig. 2I), suggesting its positive role in regulating fruit enlargement. Gene expression analysis in seven different melon cultivars (E1, E5, E6, E7, B5, B6, B7) [34] revealed significantly higher *CmYAB2* expression in elongated-fruits (large vertical diameter/high shape index) than round-fruits (small vertical diameter/low shape index) (Fig. 2J-M). This correlation analysis demonstrated positive relationships between *CmYAB2* expression and both vertical diameter (*r* = 0.94, *p* < 0.05; Fig. 2N) and fruit shape index (*r* = 0.87, *p* < 0.05; Fig. 2P), but no significant correlation with horizontal diameter (Fig. 2O).

### Transcriptome profiling of CmYAB2 downstream genes

**Fig. 3:**
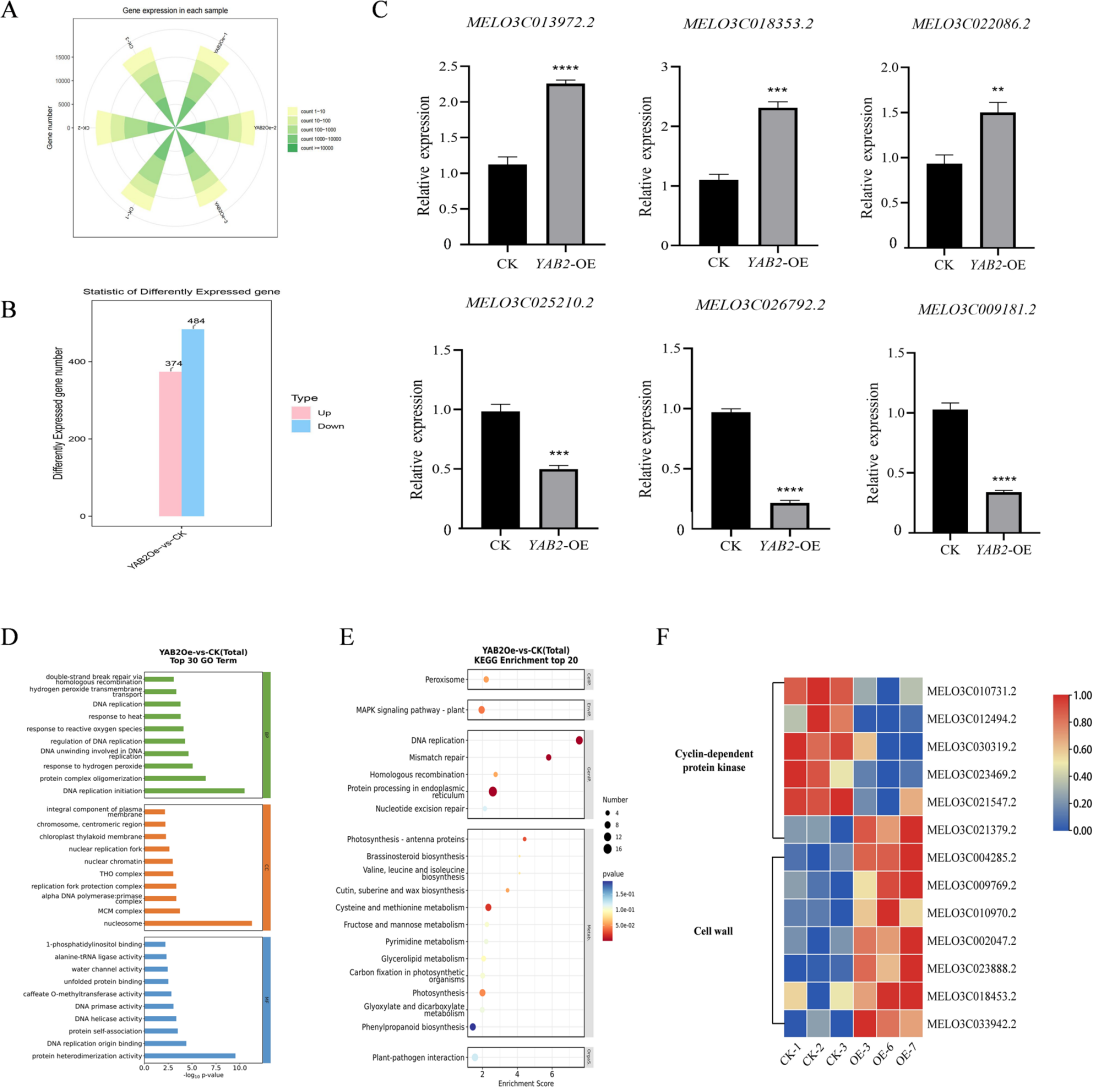
Transcriptomic profiling of *CmYAB2*-OE. **A** Transcript detection statistics. Histogram showing expressed genes in WT (1–3) and *CmYAB2*-OE (OE1-3) fruit pulp. Bars represent total genes per sample (left axis), segmented by expression magnitude. **B** The horizontal axis represents each comparison group and the vertical axis represents the number of differentiated genes. **C** RT-qPCR analysis of six significant differentially expressed genes. **D** GO enrichment analyses of top 30 significant terms. Categories are grouped into Biological Process (BP), Cellular Component (CC), and Molecular Function (MF). Bars represent -log₁₀(p-value) for terms with ≥ 5 genes. **E** KEGG enrichment (top 20). Bubble size indicates gene count; color gradient reflects significance (red: most significant). Enrichment score = -log₁₀(p-value). **F** Heatmap of differentially expressed cell cycle regulators (cyclin-dependent kinases) and cell wall development genes

To determine the potential downstream target gene of *CmYAB2* regulating melon fruit size, we performed transcriptome sequencing of the *CmYAB2* transgenic lines and WT. Transcriptome profiling detected ~ 15,000 expressed genes per biological replicate (Fig. 3A). Comparative analysis identified 858 differentially expressed genes (DEGs), comprising 374 significantly up-regulated and 484 down-regulated transcripts in *CmYAB2*-OE fruits relative to WT controls (Fig. 3B). Validation of six representative DEGs revealed coordinated regulation of growth pathways (Fig. 3C). Compared to WT, the expression of the auxin early response gene *SAUR32* (*MELO3C013972.2*) [41] and auxin efflux carrier component gene (*MELO3C018353.2*) were upregulated by 2 folds in the transgenic fruits. While gibberellin metabolism genes (*MELO3C025210*.*2*, *MELO3C026792.2*) and auxin transporter gene *MELO3C009181.2* were suppressed more than 2 folds. This downregulation of GA biosynthetic components aligns with conserved YABBY-mediated fruit size control by gibberellin signaling, as reported for tomato *SlCRCa* [28, 42]. At the same time, the cell division regulator *Cyclin-B1-2* (*MELO3C022086.2*) showed 1.6-fold induction (Fig. 3C). Go ontology analysis showed three dominant mechanisms (Fig. 3D). Biological processes featured pronounced DNA replication initiation, indicating enhanced cell proliferation. Cellular components highlighted nucleosome enrichment, implicating chromatin remodeling. Molecular functions emphasized protein heterodimerization activity, suggesting regulatory complex formation. KEGG analysis further showed DEG concentration in endoplasmic reticulum protein processing pathways (Fig. 3E). Cell cycle regulators displayed complex modulation, with most cyclin-dependent kinases downregulated - except *MELO3C021379.2* which was induced. Notably, seven cell wall development genes were upregulated (Fig. 3F), suggesting promoted cell wall loosening during fruit expansion.

### CmYAB2 *interacts with* CmYAB5 *transcription factor*

**Fig. 4:**
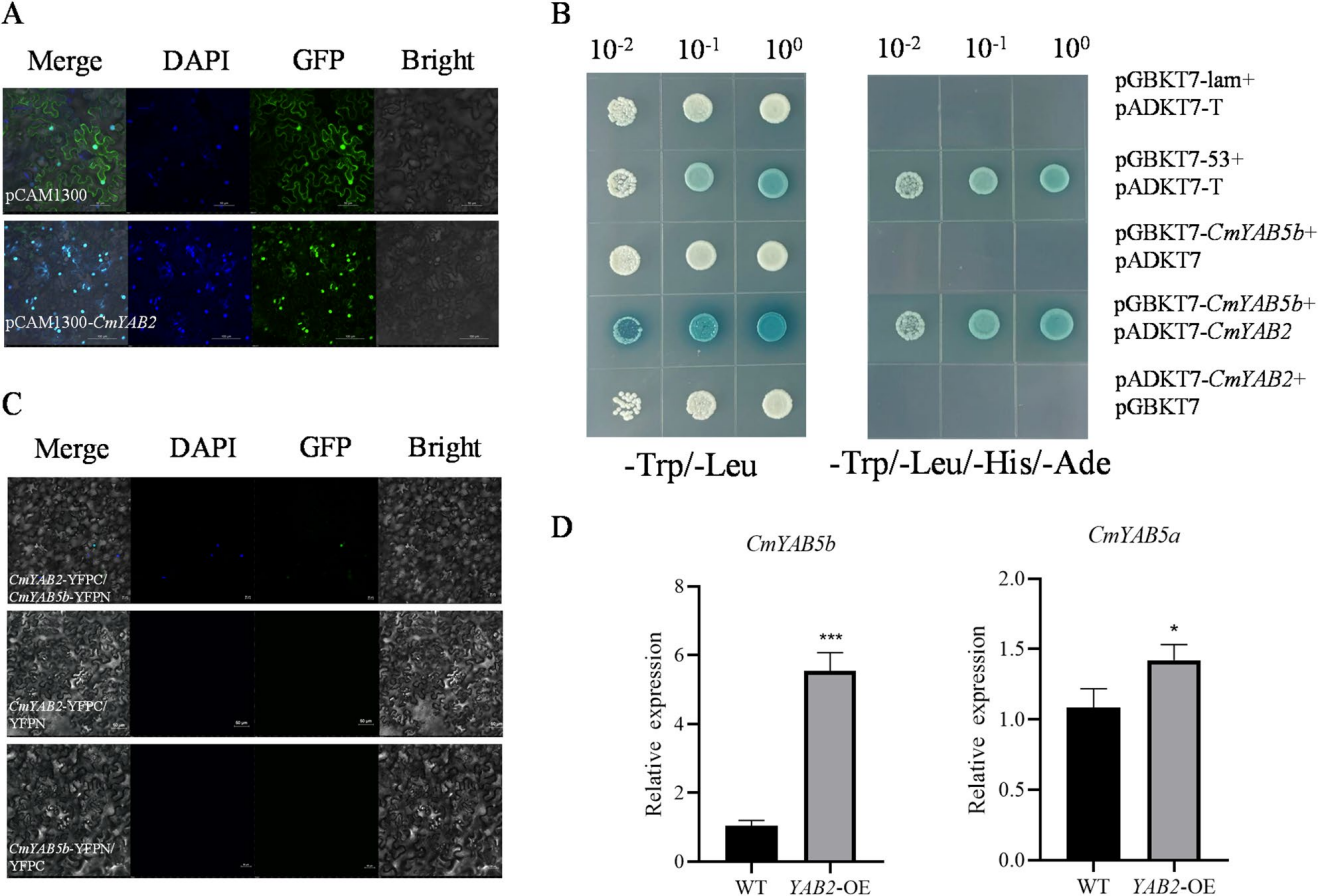
CmYAB2 interacts with CmYAB5 and specifically binds to the *CmSUN25-26-27c* promoter. **A** Subcellular localization showed that CmYAB2 protein is localized the cell nucleus of tobacco leaves. Merge, Overlapping images of the three fields. DAPI, the excitation field of blue fluorescence. GFP, the excitation field of green fluorescence. Bright, light field. **B** The yeast two-hybrid experiment verified the interaction between CmYAB2 and CmYAB5. pGBKT7-53 + pGADT7-T was used as the positive control. pGBKT7-lam + pGADT7-T, pGBKT7-*CmYAB5* + pGADT7 and pGADT7-*CmYAB2* + pGBKT7 was used as the negative control. 10^0^, 10^− 1^, and 10^− 2^ represent different dilution concentrations of yeast. **C** CmYAB2 and CmYAB5 interact in vivo. Scale bar = 50 μm. **D** Expression levels of *CmYAB5b*, *CmYAB5a* in WT and *CmYAB2*-OE melon. * *p*-value < 0.05, *** *p*-value < 0.001

*CmYAB* family member *CmYAB5b* was identified as associating with a fruit size QTL [8]. We investigated the potential gene interactions between *CmYAB2* and *CmYAB5b*. We performed the subcellular localization by inserting the pCAMBIA-1300 vector into the tobacco leaves. Observation of the GFP florescence signal showed that CmYAB2 protein was localized in the nucleus (Fig. 4A), consistent with its transcriptional function. Yeast two-hybrid assays revealed a direct physical interaction between CmYAB2 and CmYAB5b, with no detectable self-activation (Fig. 4B). This protein-protein interaction was further validated in vivo via bimolecular fluorescence complementation (BiFC), demonstrating nuclear co-localization in plant cells (Fig. 4C). These findings establish a functional protein complex between CmYAB2 and CmYAB5b, which may have redundant role in regulating melon fruit development. The expression level of *CmYAB5b* in the *CmYAB2* overexpression plants increased by approximately 5.5 times and *CmYAB5a* increased less than 1.5 fold and showed weak difference (Fig. 4D).

### CmYAB2 *binds to the promoter of CmSUN25-26-27c and activates its expression*

**Fig. 5:**
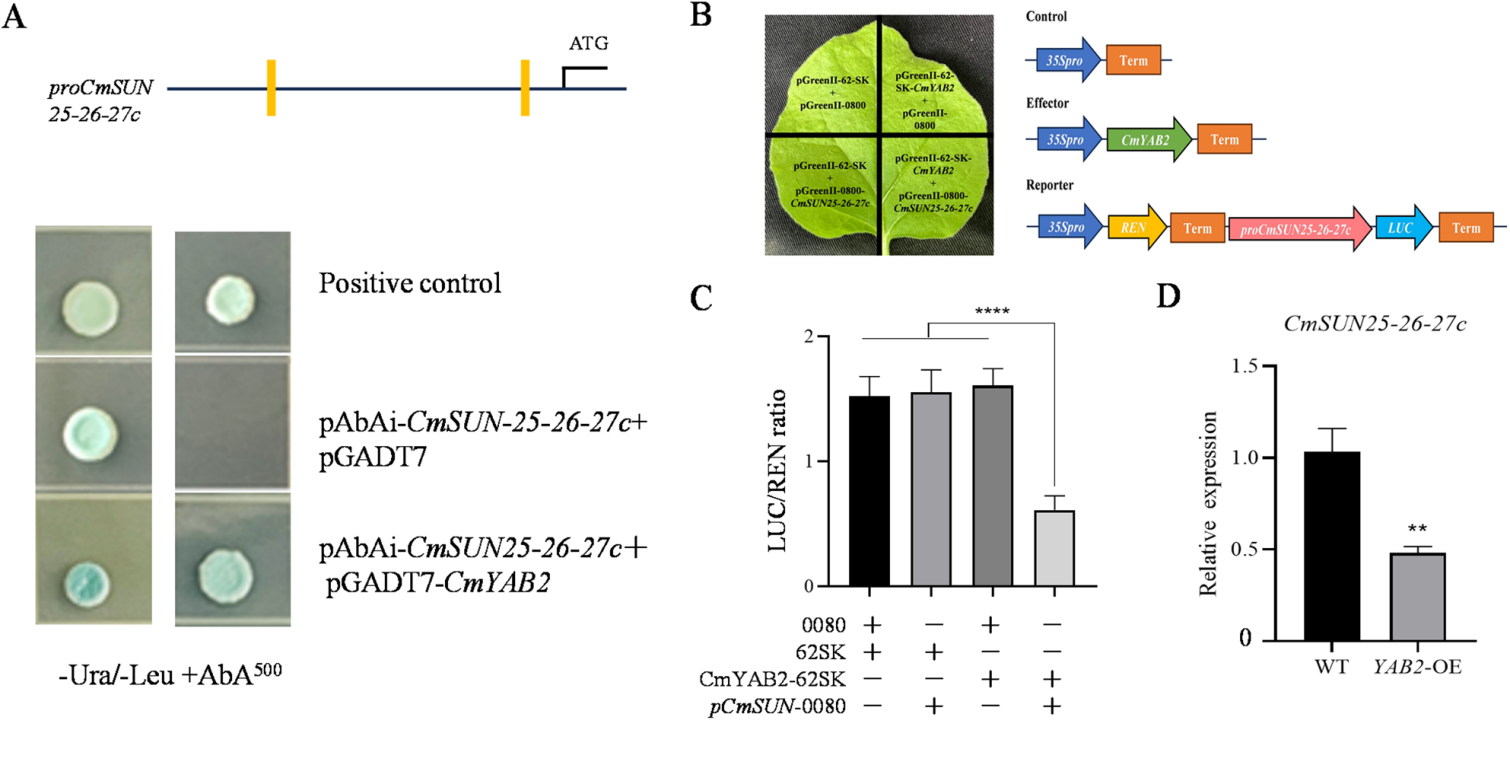
CmYAB2 binds to the promoter of *CmSUN25-26-27c* and activates its expression. **A** The distribution of element GAAAGAA in the promoter sequence of *CmSUN25-26-27c*. Yeast one-hybrid showed that CmYAB2 bind to the promoter of *CmSUN25-26-27c.* pAbAi*-CmSUN25-26-27c +* pGADT7 was used as the negative control and pAbAi*-CmSUN25-26-27c +* pGADT7-*CmYAB2* was used as the positive control. The transformation used SD/-Ura/-Leu/X-α-gal medium with an AbA concentration of 500 ng/mL. **B** Schematic diagram of dual-luciferase activity investigation. **C** LUC/REN activation assay. pGreenII-0800 + pGreenII-62-SK, pGreenII-0800 + 62SK-CmYAB2 and 0800-*CmSUN25-26-27c* + pGreenII-62-SK were the control groups. 0800-*CmSUN25-26-27c* + 62SK-*CmYAB2* was the experimental group. **** *p*-value < 0.0001. **D** Expression levels of *CmSUN25-26-27c* in WT and transgenic plants. ** *p*-value < 0.01

In melon, *CmSUN25-26-27c* plays an important role in regulating melon fruit size [33]. We identified the functional linkage of *CmSUN25-26-27c* with *CmYAB2* transcription factor. Yeast one-hybrid experiment showed that CmYAB2 could directly bind to the *cis*-element (GAAAGAA) at *CmSUN25-26-27c* promoter (Fig. 5A). Dual-luciferase transcriptional reporter assays verified that CmYAB2 negatively regulates *CmSUN25-26-27c* expression (Fig. 5B, C). Consistent with this repression mechanism, *CmSUN25-26-27c* expression levels were significantly downregulated (> 50%) in *CmYAB2*-overexpression lines (Fig. 5D).

## Discussion

Our comprehensive analysis identified seven YABBY transcription factors in *Cucumis melo*, suggesting a distinction between previous report of only four members in cucurbits [43]. This discrepancy may due to enhanced genome annotation capabilities, particularly in resolving the previously noted absence of YAB1 subfamily genes. In model plant Arabidopsis, AtYAB2 was classified as a vegetative-expressed gene in YABBY family [13]. In model horticultural crop tomato, *slyabby2a* mutants showed deficiency in septum development [44]. In melon, *CmYAB2* transcripts showed high accumulation in floral and fruit tissues. This evolutionary divergence highlighted lineage-specific functional specialization within the YABBY family. Functional characterization revealed that *CmYAB2* overexpression significantly enhances vertical/horizontal fruit diameters and fruit weight in melon, suggesting its positive role in regulating melon development. The fruit phenotype observed early in development, with enlarged ovaries at flowering progressing to significantly larger mature fruits, indicating *CmYAB2* acts during initial fruit set to modulate ultimate size.

Fruit size determination involves complex coordination of cell division, expansion, and hormonal signaling. Transcriptomic analysis of transgenic fruits clarified that overexpression of *CmYAB2* would trigger synergistic changes in the expression of cell division/cycle genes and auxin /GA pathway genes. For example, *Cyclin-B1-2* was upregulated and specific *CDKs* were downregulated. Induction of cell wall remodeling genes facilitating wall loosening. Expression of auxin transporters and gibberellin biosynthesis genes were altered in *CmYAB2-Oe* melon plant. The suppression in GA pathway genes mirrors *SlCRCa*-mediated regulation in tomato [28], suggesting conserved YABBY-gibberellin crosstalk across eudicots.

**Fig. 6:**
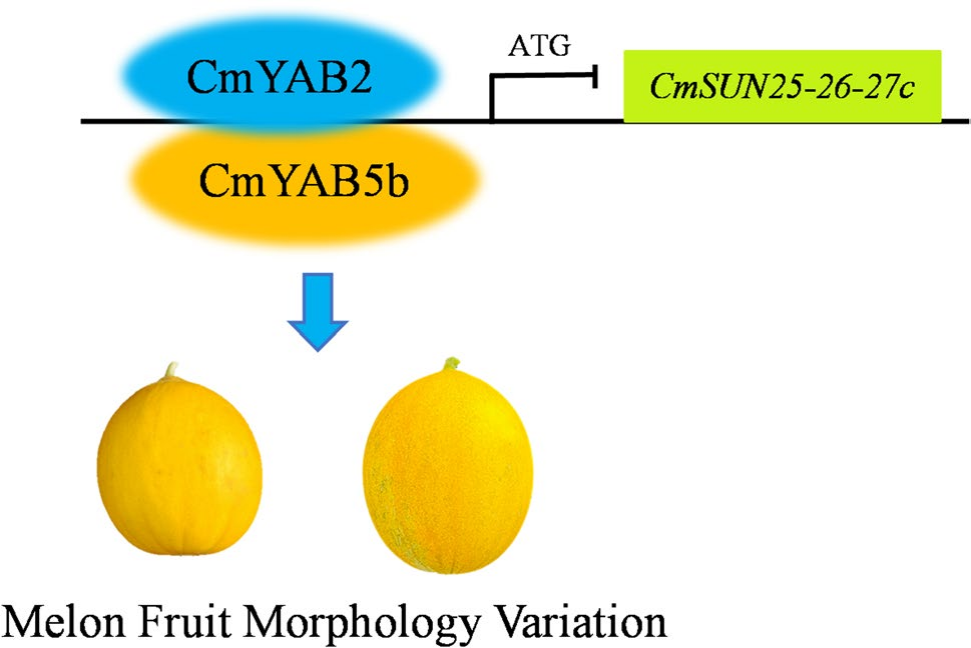
The gene regulatory model of *CmYAB2* mediating melon fruit size. The CmYAB2 protein interacts with CmYAB5 and binds to the promoter of the downstream fruit-shape regulator gene *CmSUN25-26-27c*, thereby regulating its transcription and promoting the enlargement of the fruits

The direct CmYAB2-CmYAB5b protein interaction revealed a novel regulatory complex in melon development. *SUN25-26-27a* homologs governs fruit shape determination in some Cucurbitaceae crops, such as cucumber, watermelon, and melon [45–47]. Interestingly, our findings reveal that CmYAB2 directly binds to the promoter of the *CmSUN25-26-27c* gene, inhibiting its expression. This suggests that while SUN proteins generally promote elongation in cucurbits, the CmYAB complex might finetuning this effect in melons through targeted suppression (Fig. 6). This work elucidates the molecular function of *CmYAB2* in mediating melon fruit development and provides foundational resources for molecular breeding aimed at enhancing melon yield traits.

## Conclusion

In conclusion, overexpression of *CmYAB2* leads to enhanced expansion of melon ovaries at anthesis and mature fruits, significantly increasing their vertical to horizontal diameter ratio. Protein interaction studies confirm that the CmYAB2-CmYAB5b complex suppresses the expression of the downstream *CmSUN25-26-27c* gene. This *CmYAB2*-mediated gene pathway coordinates fruit size determination, substantially advancing our understanding of YABBY transcription factor networks in melon morphogenesis and providing new targets for melon breeding. 

## Supplementary Information

Supplementary Material 1: Table S1: Primers sequences. Table S2: The information of CmYAB genes. Table S3: Transcriptome differentially expressed genes.

Supplementary Material 2: Supplemental Figure1: Sequence alignment of melon CmYAB genes.

## Data Availability

Publicly available in a repository: The original RNA-Seq sequencing data has been deposited in the NCBI sequence read archive (PRJNA1282919) and are available at the following URL: http://www.ncbi.nlm.nih.gov/sra/?term=PRJNA1282919.
